# Supplementary material for: Induced abortion in Africa: A systematic review and meta-analysis
Source: PLoS One. 2024 May 7;19(5):e0302824. doi: 10.1371/journal.pone.0302824 (PMC11075855; doi:10.1371/journal.pone.0302824)
Supplement: S2 Table — (DOCX) [file pone.0302824.s002.docx]

**S2 Table. Newcastle-Ottawa Quality Assessment Scale.**

| **Authors** | **Selection** | | | | **Comparability** | **Outcome** | | **Total score** |
| --- | --- | --- | --- | --- | --- | --- | --- | --- |
|  | Representativeness s (1) | Sample size (1) | Non-respondents (1) | Ascertainment of the exposure (risk factor) (2) | The subjects in different outcome groups are comparable, based on the study design or analysis. confounding factors are controlled (2) | Assessment of the outcome (2) | Statistical test (1) |  |
| Sahile AT et al. [22] | 1 | 1 | 1 | 2 | 1 | 1 | 1 | 8 |
| Mitiku S et al. [24] | 1 | 1 | 1 | 2 | 1 | 1 | 1 | 8 |
| Gebeyehu D et al. [25] | 1 | 1 | 1 | 2 | 1 | 2 | 1 | 9 |
| Meseret G. et al. [23] | 1 | 1 | 1 | 1 | 2 | 2 | 1 | 9 |
| Denberu B et al. [26] | 1 | 1 | 1 | 1 | 1 | 1 | 1 | 7 |
| Abebe M. et al. [27] | 1 | 1 | 1 | 1 | 1 | 2 | 1 | 8 |
| Megersa et al. [28] | 1 | 1 | 1 | 1 | 1 | 1 | 1 | 7 |
| Tesfaye G. et al. [29] | 1 | 1 | 1 | 1 | 1 | 1 | 1 | 7 |
| Tesfaye B et al. [30] | 1 | 1 | 1 | 1 | 1 | 2 | 1 | 8 |
| Gelaye et al. [31] | 1 | 1 | 1 | 1 | 1 | 2 | 1 | 8 |
| Megersa A. et al. [32] | 1 | 1 | 1 | 1 | 2 | 1 | 1 | 8 |
| Bekele D et al. [33] | 1 | 1 | 1 | 1 | 1 | 1 | 1 | 7 |
| Senbeto E. et al. [34] | 1 | 1 | 1 | 2 | 1 | 2 | 1 | 9 |
| Zeleke AM et al. [35] | 1 | 1 | 1 | 1 | 1 | 2 | 1 | 8 |
| Jamie H.A. et al. [36] | 1 | 1 | 1 | 2 | 1 | 2 | 1 | 9 |
| Nigussie et al. [37] | 1 | 1 | 1 | 1 | 1 | 2 | 1 | 8 |
| Lentiro et al. [38] | 1 | 1 | 1 | 2 | 1 | 1 | 1 | 8 |
| Bell S.O. et al. [39] | 1 | 1 | 1 | 2 | 1 | 2 | 1 | 9 |
| Ilboudo et al. [40] | 1 | 1 | 1 | 1 | 1 | 1 | 1 | 7 |
| Fatusi A, et al. [21] | 1 | 1 | 1 | 2 | 2 | 1 | 1 | 9 |
| Geelhoed DW. et al. [41] | 1 | 1 | 1 | 2 | 1 | 2 | 1 | 9 |
| Mote C.V. et al. [42] | 1 | 1 | 1 | 1 | 1 | 2 | 1 | 8 |
| Klutsey EE et al. [43] | 1 | 1 | 1 | 1 | 1 | 1 | 1 | 7 |
| Ahiadeke C. [44] | 1 | 1 | 1 | 2 | 1 | 2 | 1 | 9 |
| Baruwa OJ. et al. [45] | 1 | 1 | 1 | 1 | 2 | 2 | 1 | 9 |
| Adjei et al. [46] | 1 | 1 | 1 | 2 | 1 | 2 | 1 | 9 |
| Simmelink AM et al. [47] | 1 | 1 | 1 | 1 | 1 | 2 | 1 | 8 |
| Lugaliki AD. [48] | 1 | 1 | 1 | 1 | 1 | 1 | 1 | 7 |
| Mohamed et al. [49] | 1 | 1 | 1 | 2 | 1 | 2 | 1 | 9 |
| Okereke CI. [50] | 1 | 1 | 1 | 1 | 1 | 1 | 1 | 7 |
| Okonofua FE. et al. [51] | 1 | 1 | 1 | 1 | 1 | 1 | 1 | 7 |
| Obiyan et al. [20] | 1 | 1 | 1 | 1 | 1 | 1 | 1 | 7 |
| Murray N. et al. [52] | 1 | 1 | 1 | 1 | 1 | 1 | 1 | 7 |
| Okonofua F. et al. [53] | 1 | 1 | 1 | 2 | 1 | 2 | 1 | 9 |
| Bankole A. et al. [54] | 1 | 1 | 1 | 1 | 2 | 2 | 1 | 9 |
| Ajayi et al. [55] | 1 | 1 | 1 | 2 | 1 | 2 | 1 | 9 |
| Keogh S.C. et al. [56] | 1 | 1 | 1 | 2 | 1 | 2 | 1 | 9 |
| Kimbwereza FA et al. [57] | 1 | 1 | 1 | 1 | 1 | 1 | 1 | 7 |
| Mamboleo N. [58] | 1 | 1 | 1 | 1 | 1 | 1 | 1 | 7 |
| Prada E. et al. [59] | 1 | 1 | 1 | 2 | 1 | 2 | 1 | 9 |
| Ndari G. [60] | 1 | 1 | 1 | 1 | 1 | 1 | 1 | 7 |
| Polis C.B. et al. [61] | 1 | 1 | 1 | 2 | 1 | 2 | 1 | 9 |
| Frederico et al. [62] | 1 | 1 | 1 | 1 | 2 | 2 | 1 | 9 |
| Bell SO et al. [63] | 1 | 1 | 1 | 1 | 1 | 2 | 1 | 8 |
| Levandowski BA. et al. [64] | 1 | 1 | 1 | 2 | 1 | 2 | 1 | 9 |
| Dahlbäck E. et al. [65] | 1 | 1 | 1 | 1 | 1 | 1 | 1 | 7 |
