# Supplementary material for: Induced abortion in Africa: A systematic review and meta-analysis
Source: PLoS One. 2024 May 7;19(5):e0302824. doi: 10.1371/journal.pone.0302824 (PMC11075855; doi:10.1371/journal.pone.0302824)
Supplement: Attachment — Submitted filename: Response to Reviewers.docx [file pone.0302824.s005.docx]

**Date: April 06, 2024**

**Subject:** Response to editors, and reviewers for the revised manuscript submission.

**Title:** Induced abortion in Africa: a systematic review and meta-analysis; [PONE-D-23-21853]

**Dear Editor and Reviewers,**

We would like to thank the editor and reviewers for their time and expertise in reviewing our manuscript. your valuable input has undoubtedly contributed to the enhancement of our manuscript. We appreciate your time and effort in reviewing the previous version and considering our responses to your comments. We are glad that the revisions we made in response to your previous comments have addressed your concerns and met your expectations. We have carefully considered and incorporated your suggestions into the manuscript to improve its quality, clarity, and scientific rigor. Once again, we sincerely appreciate your positive response and we are grateful for your thorough evaluation of our manuscript.

**Additional Editor Comments:**

Thank you for the opportunity to manage the submission of this current manuscript. I had the opportunity to review the current and latest versions and the comments from the reviewers. The manuscript is improved and it is very well-written. In order to improve clarity and reproducibility, I suggest making the data extraction process and objectives related to the subgroup analyses done in the meta-analysis clear: unintended pregnancy, unmarried women, educational status, and substance abuse. It was not clear enough whether such analysis was planned before conducting the systematic review or whether it was conducted after retrieving papers and looking at the available data. Data-driven analysis can introduce many biases. Make it clear whether the study protocol has been previously registered or published.

**Response:** We appreciate your careful reading of our manuscript and your insightful comments. We acknowledge the concerns expressed about the data extraction process and objectives related to the subgroup analyses, publication biases, and study protocol, as well as your recommendations for enhancing clarity and reproducibility.

Regarding the data extraction process, it is stated explicitly on pages 5-6 (lines 106–112) that "The data were separately extracted by three authors using a structured method of data collection." the objective of subgroup analyses is to compare these overall estimates across groups and determine whether the considered grouping helps us explain some of the observed between-study heterogeneity, and the studies were grouped based on the study area and study year, and an overall effect size was computed for each group, This is covered in detail on page number 12 (lines 204–215). "Unintended pregnancy, unmarried women, educational status, and substance abuse" are not subgroup analyses; rather, they are characteristics that significantly predict the likelihood of an induced abortion.

Regarding publication bias, we have reported the results of the funnel plot, Egger's test, and trim and fill test, all of which showed no significant publication bias; this suggests that the results of our meta-analysis are not unduly influenced by the selective publication of studies based on their outcomes. Page number 11-12 (lines 189-202).

We regret to notify you, nonetheless, that a study protocol was not registered or published for this research. We recognize that in order to improve transparency and reduce biases, pre-registration or publication of the study protocol is crucial. We have taken many precautions to reduce potential biases by adhering to established guidelines for systematic reviews and meta-analyses, following a rigorous methodology, and providing a detailed description of our methodology and analysis approach, even though the lack of a registered or published protocol is a limitation. Finally, the lack of a registered or published protocol is mentioned as a limitation for this study, on page 16 (lines 298–303) of our work. We hope to keep things transparent and let readers know about this restriction by doing this. Once again, we appreciate your thorough review and constructive feedback.
