# Supplementary material for: Induced abortion in Africa: A systematic review and meta-analysis
Source: PLoS One. 2024 May 7;19(5):e0302824. doi: 10.1371/journal.pone.0302824 (PMC11075855; doi:10.1371/journal.pone.0302824)
Supplement: Attachment — Submitted filename: Reviewer Comments for authors.docx [file pone.0302824.s003.docx]

Reviewer Comments for authors

1. Is the manuscript technically sound, and do the data support the conclusions?

Yes, the manuscript is technically sound, as it follows a systematic review and meta-analysis method to estimate the pooled prevalence of induced abortion and its associated factors in Africa. The data support the conclusions, as they show that the prevalence of induced abortion is high and varies by country and year of study.

2. Has the statistical analysis been performed appropriately and rigorously?

Yes

The statistical analysis has been performed appropriately and rigorously, as the authors use a systematic review and meta-analysis method to combine the results of different studies on the prevalence of induced abortion and its associated factors in Africa. The authors use software called STATA 14, which is a widely used and reliable tool for statistical analysis. They also use funnel plot and Egger regression test to assess the potential publication bias, which is the tendency of published studies to report more favorable or significant results than the true effect. They perform sub-group analysis by country and year of study to explore the sources of heterogeneity and to compare the results across different settings and time periods. The authors follow the standard and rigorous procedures for conducting a meta-analysis and reporting their findings.

3. Have the authors made all data underlying the findings in their manuscript fully available?

Yes

4. Is the manuscript presented in an intelligible fashion and written in Standard English?

Yes, The manuscript is presented in an intelligible fashion and written in Standard English, as it follows a clear and logical structure of background, methods, results, and conclusion.
